# Supplementary material for: ERBB2 establishes the timing of progesterone priming required for uterine receptivity
Source: bioRxiv. 2026 Sep 16:2026.09.14.751518. Preprint. [Version 1] doi: 10.64898/2026.09.14.751518 (PMC13596335; doi:10.64898/2026.09.14.751518)
Supplement: Supplementary file 1 [file NIHPP2026.09.14.751518v1-supplement-1.pdf]

bioRxiv preprint doi: https://doi.org/10.64898/2026.09.14.751518; this version posted September 16, 2026. The copyright holder for this preprint (which was not certified by peer review) is the author/funder, who has granted bioRxiv a license to display the preprint in perpetuity. It is made available under a CC-BY 4.0 International license.

Supplementary information:

ERBB2 establishes the timing of progesterone priming required for uterine receptivity

Bo Li[1,2,†], Chen Zhang[3,4†], Amanda Dewar[1,2], Xiaoli Liu[5,6], Wenbo Deng[7,8], Hongbo Qi[3,4*], Sudhansu K. Dey[1,2,*] & Xiaofei Sun[1,2,*]

[1]Reproductive Sciences Center, Division of Developmental Biology, Cincinnati Children’s Hospital Medical Center, Cincinnati, OH 45229, USA

[2]College of Medicine, University of Cincinnati, Cincinnati, OH 45221, USA

[3]Department of Obstetrics and Gynecology, The First Affiliated Hospital of Chongqing Medical University, Chongqing 400016, China

[4]Chongqing Key Laboratory of Maternal and Fetal Medicine, The First Affiliated Hospital of Chongqing Medical University, Chongqing 400016, China

[5]Department of Obstetrics and Gynecology, Women and Children's Hospital of Chongqing Medical University, Chongqing, 401147, China

[6]Department of Obstetrics and Gynecology, Chongqing Health Center for Women and Children, Chongqing, 401147, China

[7]Fujian Provincial Key Laboratory of Reproductive Health Research, Department of Obstetrics and Gynecology, The First Affiliated Hospital of Xiamen University, School of Medicine, Xiamen University, Xiamen, Fujian 361102, China.

[8]State Key Laboratory of Vaccines for Infectious Diseases, Xiang An Biomedicine Laboratory, School of Medicine, Xiamen University, Xiamen, Fujian, China.

[†]These authors contributed equally to this work.

*Corresponding authors: Hongbo Qi (qihongbo@cqmu.edu.cn), Sudhansu K. Dey (sk.dey@cchmc.org) and Xiaofei Sun (xiaofei.sun@cchmc.org)

bioRxiv preprint doi: https://doi.org/10.64898/2026.09.14.751518; this version posted September 16, 2026. The copyright holder for this preprint (which was not certified by peer review) is the author/funder, who has granted bioRxiv a license to display the preprint in perpetuity. It is made available under a CC-BY 4.0 International license.

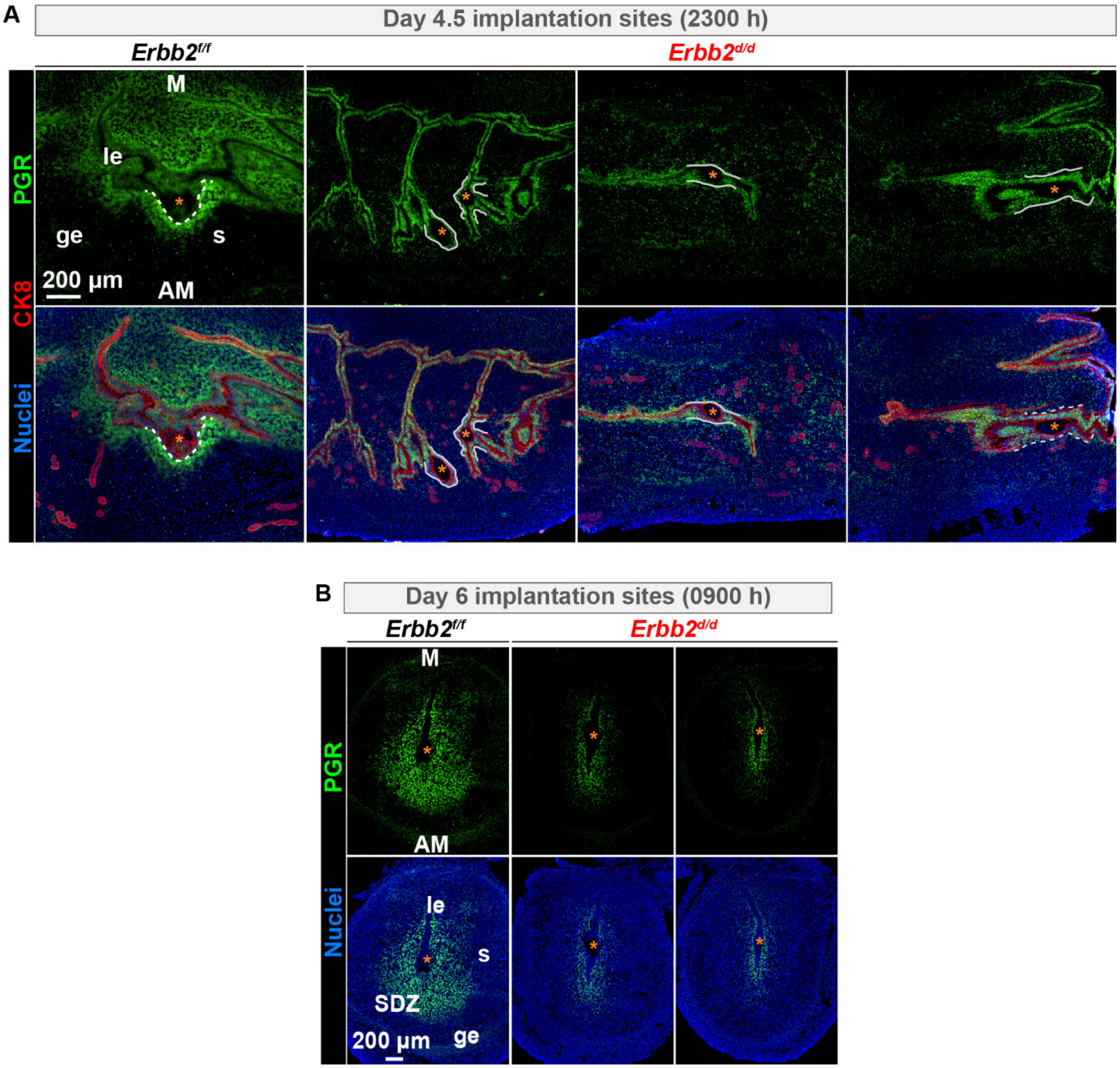

Supplemental Figure 1. PGR expression in *Erbb2$^{f/f}$* and *Erbb2$^{d/d}$* uteri on day 2, day 3, day 4.5, and day 6 of pregnancy. (**A**) Immunofluorescence of PGR and CK8 in implantation sites at day 4.5 of pregnancy (2300 h) from *Erbb2$^{f/f}$* (n = 3) and *Erbb2$^{d/d}$* (n = 3) uteri. Scale bar, 200 μm. (**B**) Immunofluorescence of PGR in implantation sites at 0900 h on day 6 from *Erbb2$^{f/f}$* (n = 4) and *Erbb2$^{d/d}$* (n = 4) uteri. Scale bar, 200 μm. Asterisks indicate the location of the embryo. le luminal epithelium, ge glandular epithelium, s stroma, M mesometrial pole, AM antimesometrial pole, SDZ secondary decidual zone.

bioRxiv preprint doi: https://doi.org/10.64898/2026.09.14.751518; this version posted September 16, 2026. The copyright holder for this preprint (which was not certified by peer review) is the author/funder, who has granted bioRxiv a license to display the preprint in perpetuity. It is made available under a CC-BY 4.0 International license.

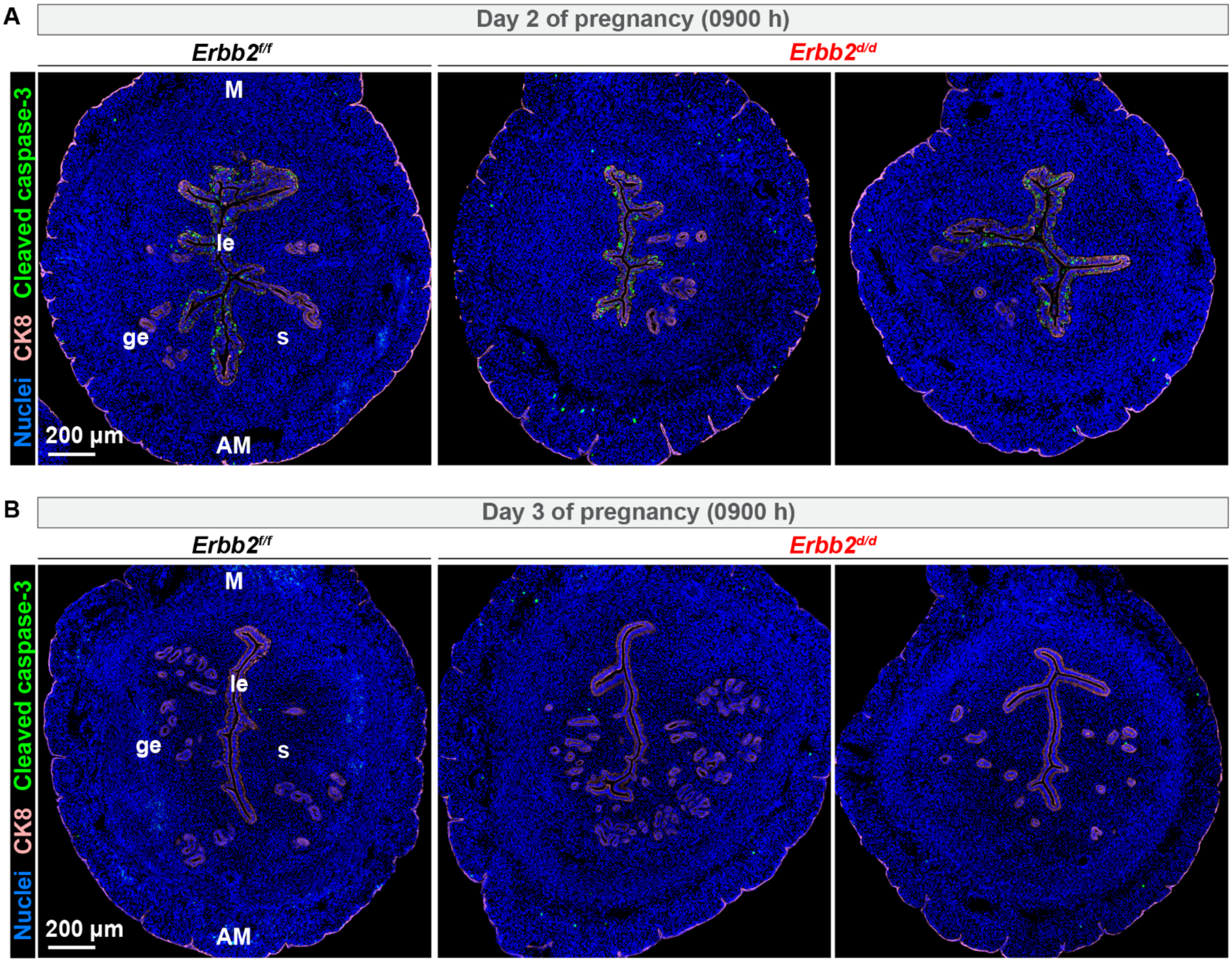

Supplemental Figure 2. Analysis of epithelial apoptosis in *Erbb2$^{d/d}$* mice on day 2 and day 3 of pregnancy. (**A** and **B**) Immunofluorescence staining of cleaved caspase-3 and CK8 from *Erbb2$^{f/f}$* and *Erbb2$^{d/d}$* mice on day 2 (**A**; n = 3 for *Erbb2$^{f/f}$*, n = 3 for *Erbb2$^{d/d}$*) and day 3 (**B**; n = 3 for *Erbb2$^{f/f}$*, n = 3 for *Erbb2$^{d/d}$*) of pregnancy (0900 h). Nuclei were counterstained with DAPI. le luminal epithelium, ge glandular epithelium, s stroma, M mesometrial pole, AM antimesometrial pole.

bioRxiv preprint doi: https://doi.org/10.64898/2026.09.14.751518; this version posted September 16, 2026. The copyright holder for this preprint (which was not certified by peer review) is the author/funder, who has granted bioRxiv a license to display the preprint in perpetuity. It is made available under a CC-BY 4.0 International license.

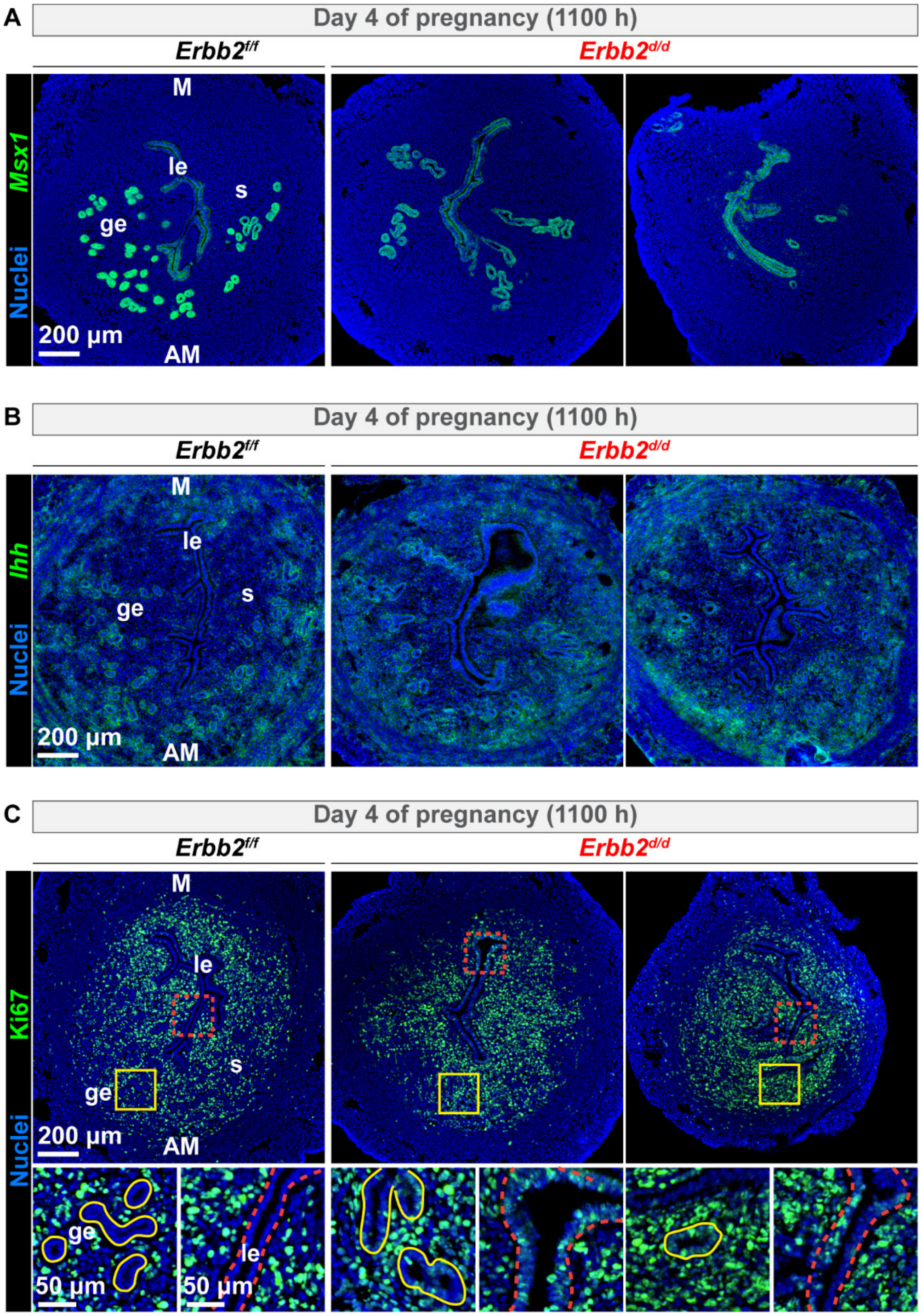

Supplemental Figure 3. *Msx1* and *Ihh* are not altered in *Erbb2$^{d/d}$* uteri on day 4 of pregnancy. (**A**) FISH of *Msx1* from *Erbb2$^{f/f}$* (n = 3) and *Erbb2$^{d/d}$* (n = 4) mice on day 4 of pregnancy (1100 h). Scale bar, 200 µm. (**B**) FISH of *Ihh* revealed comparable expression levels in *Erbb2$^{f/f}$* (n = 6) and *Erbb2$^{d/d}$* (n = 6) uteri. Scale bar, 200 µm. (**C**) Ki67 immunostaining from *Erbb2$^{f/f}$* (n = 3) and *Erbb2$^{d/d}$* (n = 4) mice on day 4 of pregnancy (1100 h). Scale bar, 200 µm; magnified scale bars: 50 µm. le luminal epithelium, ge glandular epithelium, s stroma, M mesometrial pole, AM antimesometrial pole.

bioRxiv preprint doi: https://doi.org/10.64898/2026.09.14.751518; this version posted September 16, 2026. The copyright holder for this preprint (which was not certified by peer review) is the author/funder, who has granted bioRxiv a license to display the preprint in perpetuity. It is made available under a CC-BY 4.0 International license.

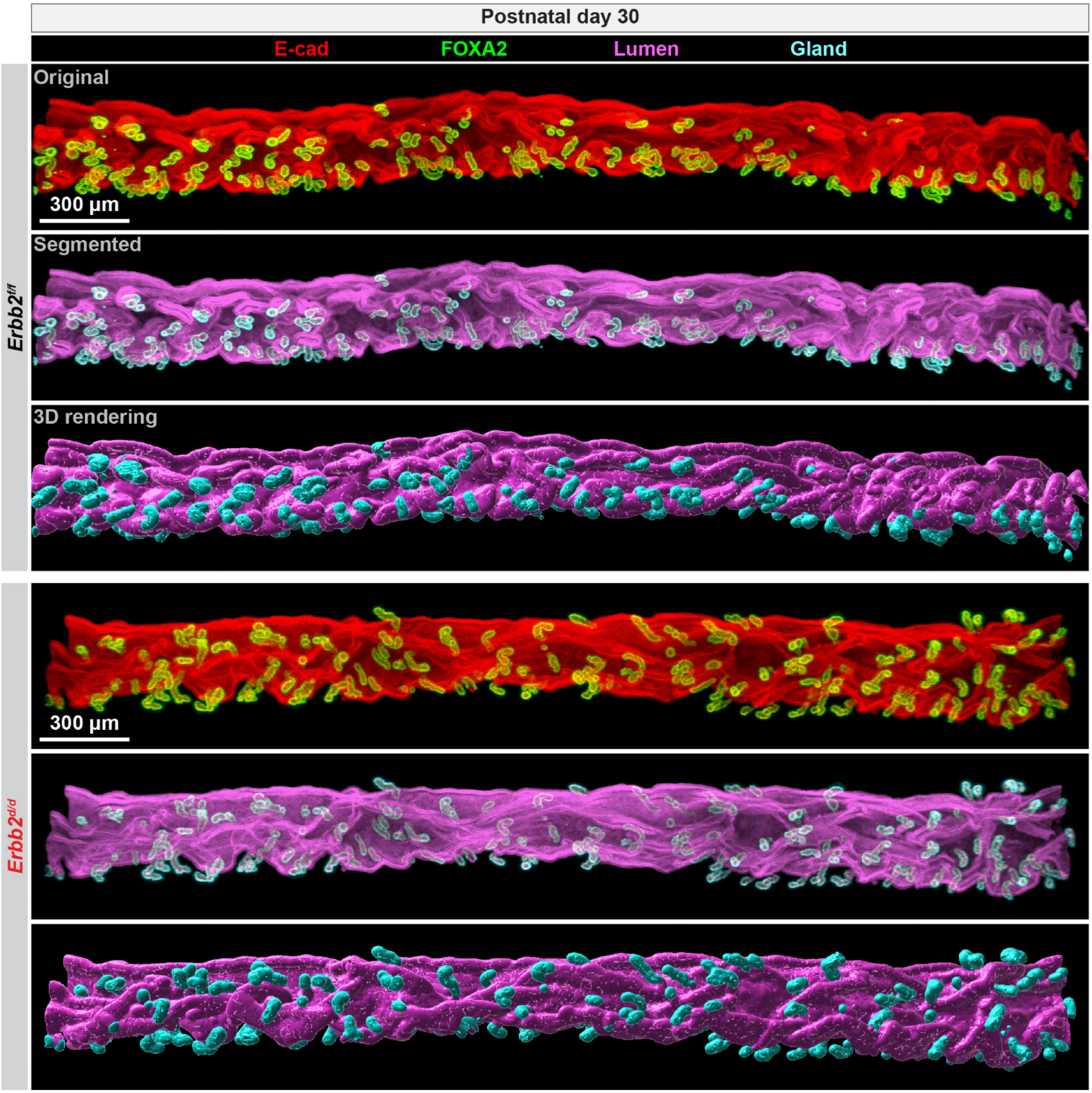

bioRxiv preprint doi: https://doi.org/10.64898/2026.09.14.751518; this version posted September 16, 2026. The copyright holder for this preprint (which was not certified by peer review) is the author/funder, who has granted bioRxiv a license to display the preprint in perpetuity. It is made available under a CC-BY 4.0 International license.

Supplemental Figure 4. 3D imaging from *Erbb2*$^{f/f}$ and *Erbb2*$^{d/d}$ females on postnatal day 30. Original double immunostaining with E-cadherin and FOXA2 and 3D rendering pictures. E-cadherin marks both luminal epithelial and glandular epithelial cells, while FOXA2 only marks glands. Scale bars, 300 μm.

bioRxiv preprint doi: https://doi.org/10.64898/2026.09.14.751518; this version posted September 16, 2026. The copyright holder for this preprint (which was not certified by peer review) is the author/funder, who has granted bioRxiv a license to display the preprint in perpetuity. It is made available under a CC-BY 4.0 International license.

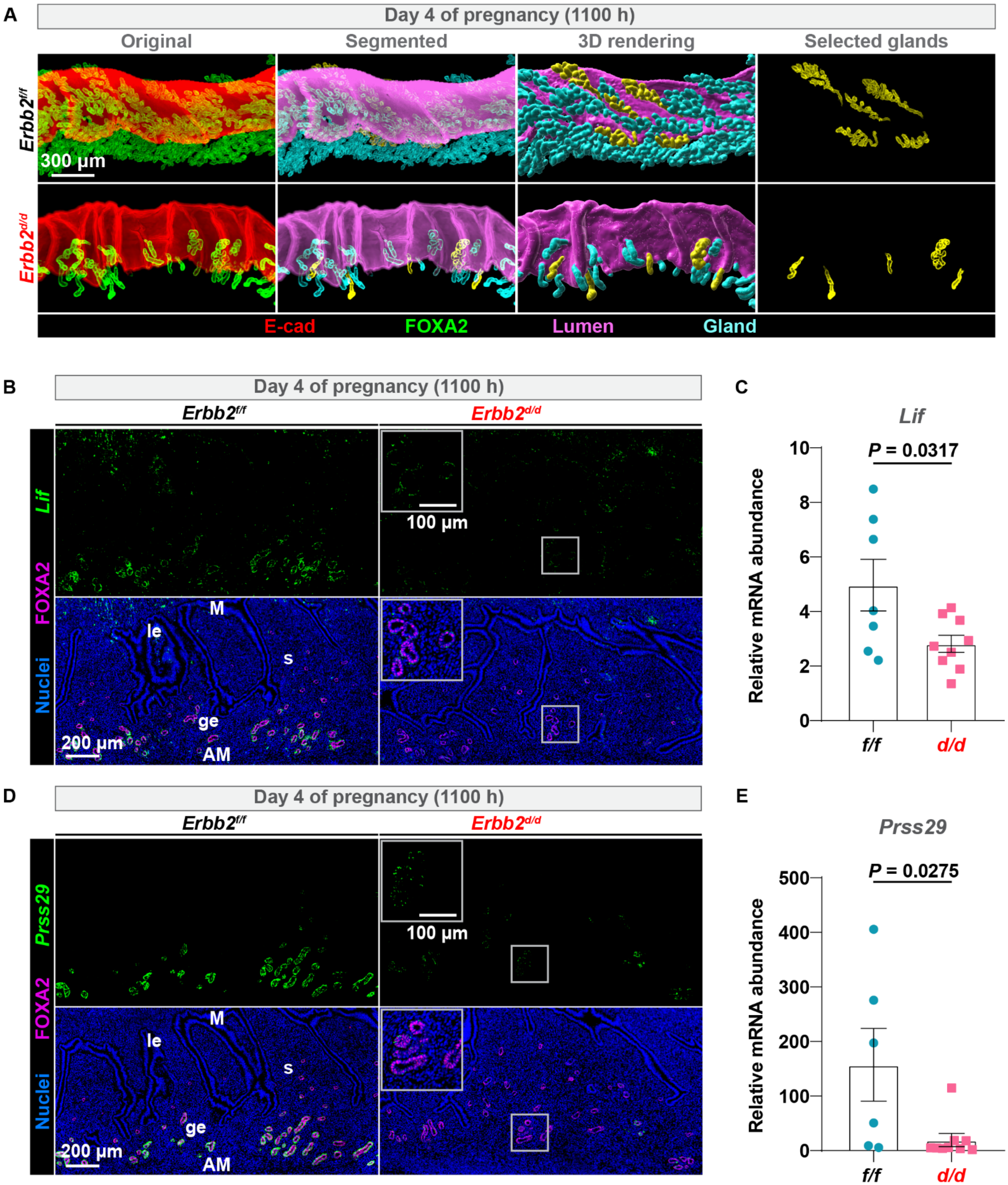

Supplemental Figure 5. Uterine gland morphology and LIF secretion are abnormal in *Erbb2$^{d/d}$* mice on day 4 of pregnancy. (**A**) 3D imaging in *Erbb2$^{f/f}$* and *Erbb2$^{d/d}$* uteri on day 4 of pregnancy at 1100 h. Scale bar, 300 µm. Original (E-cadherin and FOXA2), Segmented, and 3D rendering. le luminal epithelium, ge glandular

bioRxiv preprint doi: https://doi.org/10.64898/2026.09.14.751518; this version posted September 16, 2026. The copyright holder for this preprint (which was not certified by peer review) is the author/funder, who has granted bioRxiv a license to display the preprint in perpetuity. It is made available under a CC-BY 4.0 International license.

epithelium, s stroma, M mesometrial pole, AM antimesometrial pole. (**B**) FISH of *Lif* and Immunofluorescence of FOXA2 in *Erbb2*$^{f/f}$ (n = 6) and *Erbb2*$^{d/d}$ (n = 7) uteri on day 4 of pregnancy at 1100 h. Scale bar, 200 μm; magnified scale bar: 100 μm. (**C**) qPCR analysis of *Lif* mRNA levels in uteri from *Erbb2*$^{f/f}$ (n = 7) and *Erbb2*$^{d/d}$ (n = 9) mice. Data are presented as mean ± SEM. (**D**) FISH of *Prss29* and Immunofluorescence of FOXA2 in *Erbb2*$^{f/f}$ (n = 6) and *Erbb2*$^{d/d}$ (n = 7) uteri on day 4 of pregnancy at 1100 h. Scale bar, 200 μm; magnified scale bar: 100 μm. (**E**) qPCR analysis of *Prss29* mRNA levels in uteri from *Erbb2*$^{f/f}$ (n = 6) and *Erbb2*$^{d/d}$ (n = 9) mice. Data are presented as mean ± SEM.

bioRxiv preprint doi: https://doi.org/10.64898/2026.09.14.751518; this version posted September 16, 2026. The copyright holder for this preprint (which was not certified by peer review) is the author/funder, who has granted bioRxiv a license to display the preprint in perpetuity. It is made available under a CC-BY 4.0 International license.

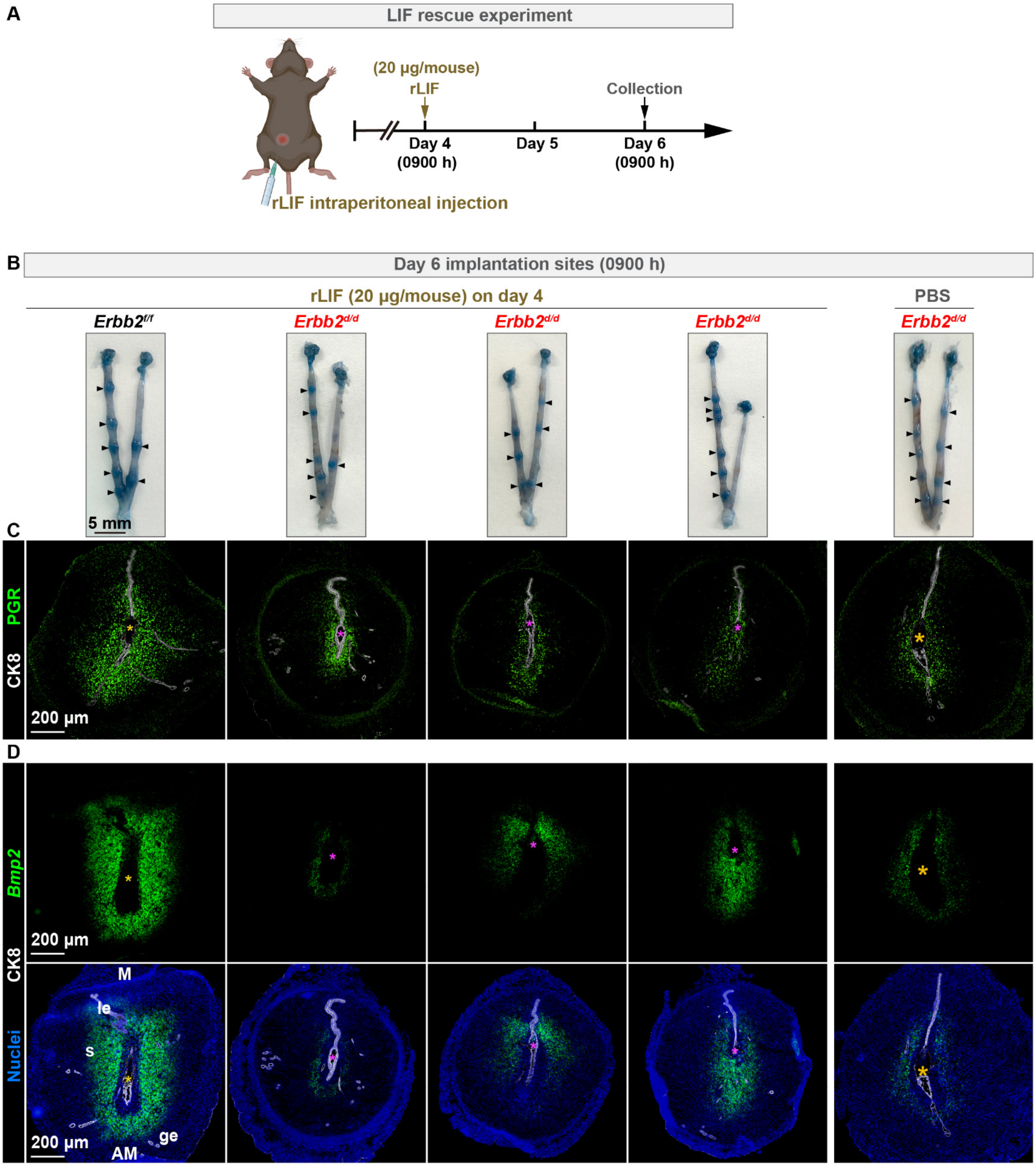

Supplemental Figure 6. rLIF supplementation partially rescue embryo implantation in *Erbb2*$^{d/d}$ mice. (**A**) Schema of experimental design. Recombinant LIF (rLIF, 20 µg/mouse) was intraperitoneally injected into *Erbb2*$^{f/f}$ and *Erbb2*$^{d/d}$ mice at 0900 h on day 4 of pregnancy, and uteri were collected at 0900 h on day 6 of pregnancy

bioRxiv preprint doi: https://doi.org/10.64898/2026.09.14.751518; this version posted September 16, 2026. The copyright holder for this preprint (which was not certified by peer review) is the author/funder, who has granted bioRxiv a license to display the preprint in perpetuity. It is made available under a CC-BY 4.0 International license.

(*Erbb2*$^{f/f}$ with LIF treatment: n = 3; *Erbb2*$^{d/d}$ with LIF treatment: n = 3; *Erbb2*$^{d/d}$ with PBS treatment: n = 3). (**B**) Representative images of implantation sites from *Erbb2*$^{f/f}$ and *Erbb2*$^{d/d}$ mice on day 6. Scale bar, 5 mm. (**C**) Immunofluorescence staining showing PGR and CK8 expression at implantation sites. Scale bar, 200 μm. (**D**) FISH of decidualization marker *Bmp2* and CK8. Scale bars, 200 μm.

bioRxiv preprint doi: https://doi.org/10.64898/2026.09.14.751518; this version posted September 16, 2026. The copyright holder for this preprint (which was not certified by peer review) is the author/funder, who has granted bioRxiv a license to display the preprint in perpetuity. It is made available under a CC-BY 4.0 International license.

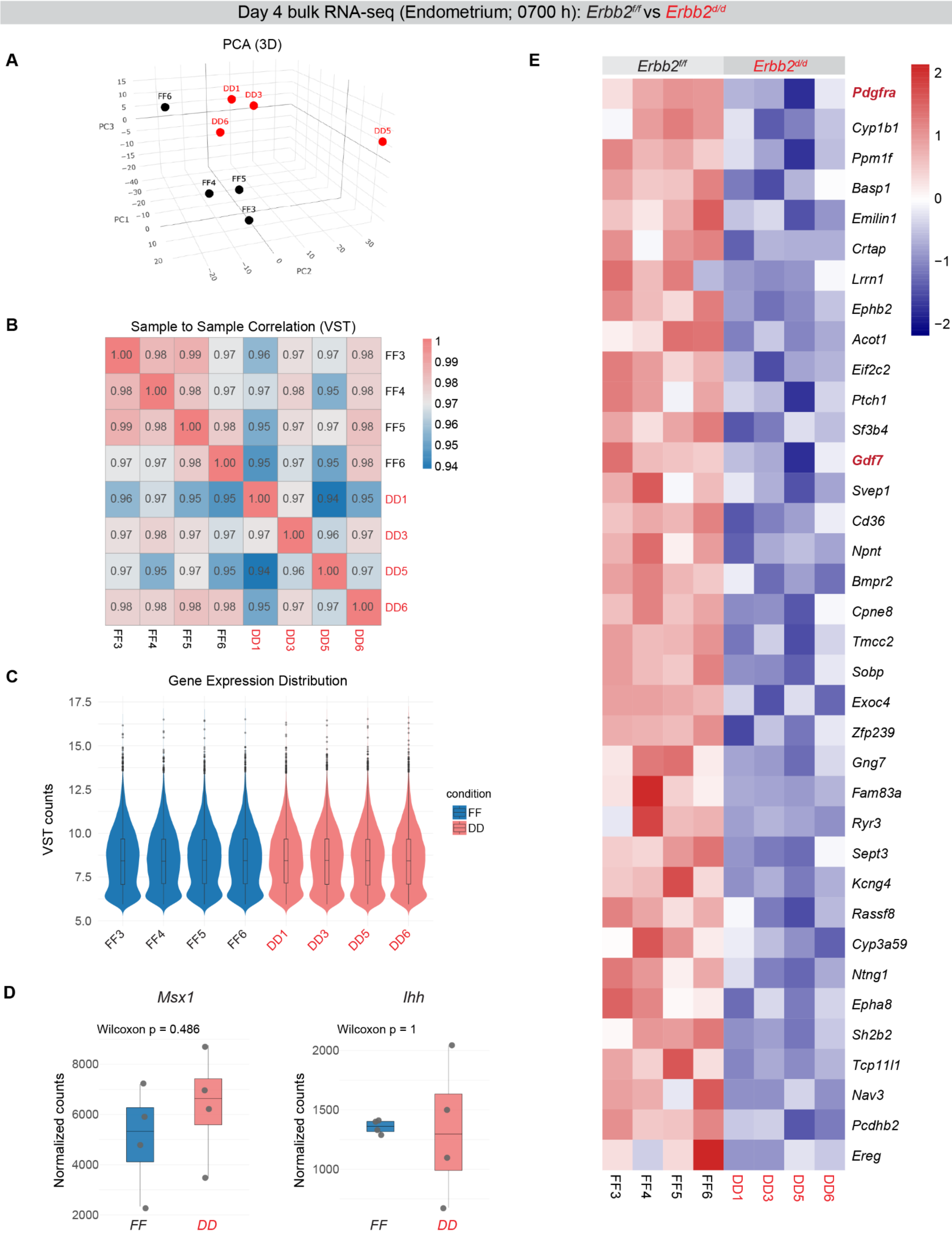

bioRxiv preprint doi: https://doi.org/10.64898/2026.09.14.751518; this version posted September 16, 2026. The copyright holder for this preprint (which was not certified by peer review) is the author/funder, who has granted bioRxiv a license to display the preprint in perpetuity. It is made available under a CC-BY 4.0 International license.

Supplemental Figure 7. Bulk RNA-seq analysis of day 4 endometrium (0700 h) reveals stromal gene alterations in *Erbb2*$^{d/d}$ uteri. (**A**) 3D principal component analysis (PCA) of bulk RNA-seq data from day 4 (0700 h) endometrial tissues of *Erbb2*$^{f/f}$ (n =4) and *Erbb2*$^{d/d}$ (n =4) mice. (**B**) Sample-to-sample correlation heatmap based on variance-stabilizing-transformed (VST) counts. (**C**) Gene expression distribution (VST counts) across biological replicates. (**D**) Normalized expression of classical uterine receptivity markers, *Msx1* and *Ihh* (Wilcoxon test). (**E**) Heatmap of significantly downregulated genes in DD samples. Color scale indicates z-score–normalized expression.

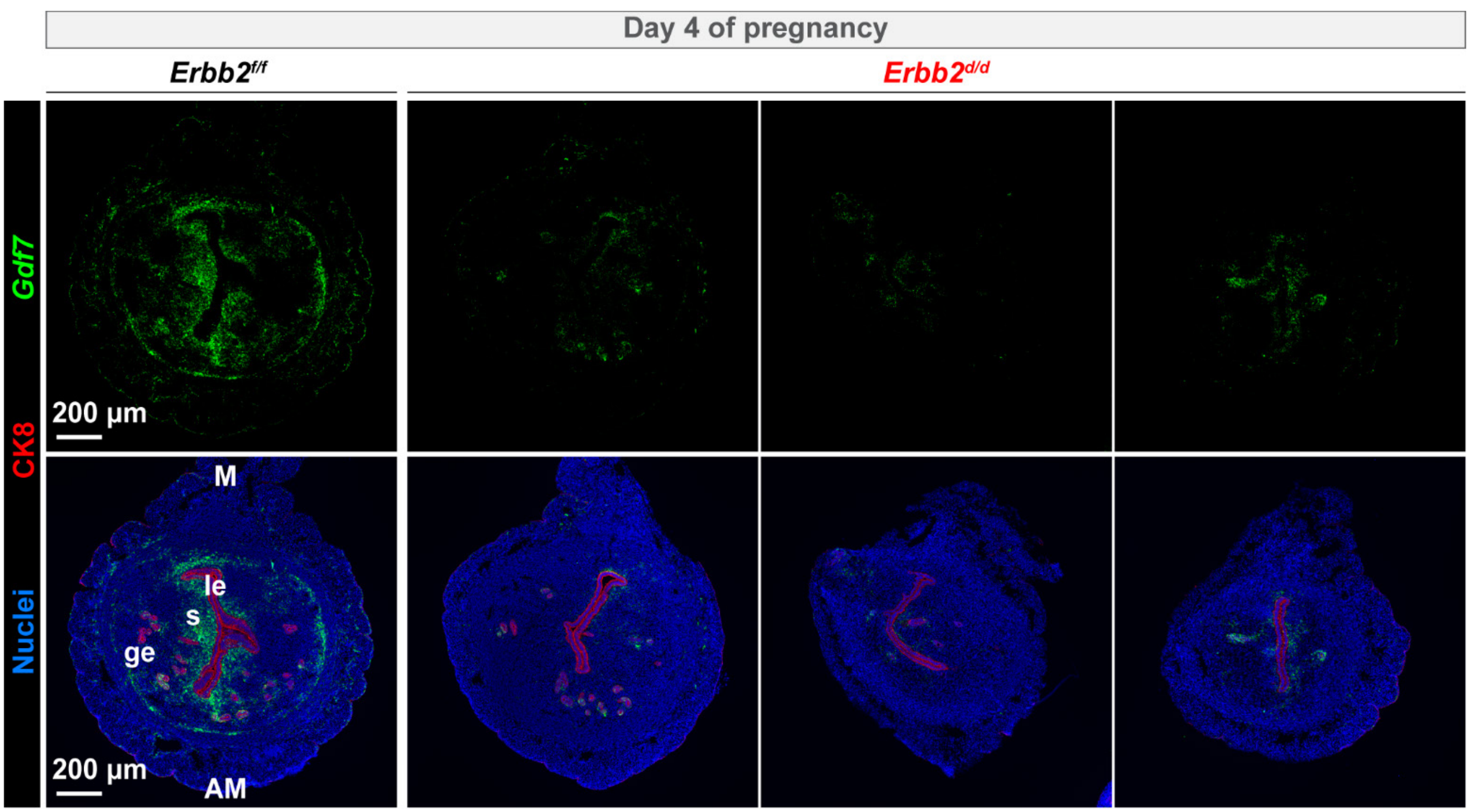

Supplemental Figure 8. Stromal *Gdf7* was reduced in *Erbb2*$^{d/d}$ mice on day 4 of pregnancy. FISH of *Gdf7* and immunofluorescence of CK8 in *Erbb2*$^{f/f}$ (n = 3) and *Erbb2*$^{d/d}$ (n = 3) uteri on day 4 of pregnancy. Scale bar, 200 µm. le luminal epithelium, ge glandular epithelium, s stroma, M mesometrial pole, AM antimesometrial pole.

bioRxiv preprint doi: https://doi.org/10.64898/2026.09.14.751518; this version posted September 16, 2026. The copyright holder for this preprint (which was not certified by peer review) is the author/funder, who has granted bioRxiv a license to display the preprint in perpetuity. It is made available under a CC-BY 4.0 International license.

Supplemental Table 1. Clinical characteristics of women included in normal and RSA samples.

	normal (n=21)	RSA (n=21)	*P*
Age (years)	26.71±2.51	27.86±3.24	0.209
Gestation age (weeks)	8.46±0.85	8.3±0.76	0.521
BMI	21.64±1.51	22.17±0.99	0.171
smoking status	None	None	
Previous unexplained pregnancy loss	0	2-4	$<$ 0.0001
Parity	0-2	0-1	0.338
Chromosomal analysis	-	normal	-

Supplemental Table 2. Sequences of primers used for qRT-PCR analyses.

Genes	Sequence (5' to 3')
ACTB -homo-F	AGAAAATCTGGCACCACACCT
ACTB -homo-R	GATAGACAGCCTGGATAGCA
ERBB2-homo-F	GTGTGCTATGGTCTGGGCAT
ERBB2-homo-R	AATGCCAGGCTCCCAAAGAT
PGR-homo-F	GTCGAGCTCACAGCGTTTCTA
PGR-homo-R	AAATTCAACACTCAGTGCCCG
